# Force-Time Differences between Ballistic and Non-Ballistic Half-Squats

**DOI:** 10.3390/sports6030079

**Published:** 2018-08-12

**Authors:** Timothy J. Suchomel, Christopher B. Taber, Christopher J. Sole, Michael H. Stone

**Affiliations:** 1 Department of Human Movement Sciences, Carroll University, Waukesha, WI 53186, USA; 2 Department of Exercise Science, Sacred Heart University, Fairfield, CT 06825, USA; taberc@sacredheart.edu; 3 Department of Health and Human Performance, The Citadel—The Military College of South Carolina, Charleston, SC 29409, USA; csole@citadel.edu; 4 Department of Exercise and Sport Sciences, Center of Excellence for Sport Science and Coach Education, East Tennessee State University, Johnson City, TN 37614, USA; stonem@etsu.edu

**Keywords:** partial squat, intent, resistance training, strength

## Abstract

The purpose of this study was to examine the force-time differences between concentric-only half-squats (COHS) performed with ballistic (BAL) or non-ballistic (NBAL) intent across a range of loads. Eighteen resistance-trained men performed either BAL or NBAL COHS at 30%, 50%, 70%, and 90% of their one repetition maximum (1RM) COHS. Relative peak force (PF) and relative impulse from 0–50 ms (Imp50), 0–90 ms (Imp90), 0–200 ms (Imp200), and 0–250 ms (Imp250) were compared using a series of 2 × 4 (intent × load) repeated measures ANOVAs with Bonferroni post hoc tests. Cohen’s d effect sizes were calculated to provide measures of practical significance between the BAL and NBAL COHS and each load. BAL COHS produced statistically greater PF than NBAL COHS at 30% (d = 3.37), 50% (d = 2.88), 70% (d = 2.29), and 90% 1RM (d = 1.19) (all *p* < 0.001). Statistically significant main effect differences were found between load-averaged BAL and NBAL COHS for Imp90 (*p* = 0.006, d = 0.25), Imp200 (*p* = 0.001, d = 0.36), and Imp250 (*p* < 0.001, d = 0.41), but not for Imp50 (*p* = 0.018, d = 0.21). Considering the greater PF and impulse observed during the BAL condition, performing COHS with BAL intent may provide a favorable training stimulus compared to COHS performed with NBAL intent.

## 1. Introduction

A wide variety of training methods may be implemented when training and developing maximal and explosive strength characteristics (e.g., peak force (PF), impulse, rate of force development, and power output) [1,2]. One such method for training the lower body includes prescribing partial range of motion exercises, such as the half squat or quarter squat. Implementing these exercises allows individuals to use supramaximal loads (i.e., loads in excess of their one repetition maximum (1RM) that cannot be lifted through a full range motion), which may allow for enhancements in maximal force production via reduced neuromuscular inhibition [3]. Moreover, when supramaximal loads are lifted from a stationary position (e.g., off of safety pins or boxes), the lifter is forced to overcome its static inertia, which may benefit both impulse and rate of force development characteristics [4]. In fact, previous research displayed greater increases in maximal strength and early force-time characteristics when implementing partial range of motion squat variations with full range motion squats compared to only using full range of motion squats [5]. Further research displayed improvements in squat strength, vertical jump height, and 40-yard sprint speed in highly-trained men following 16 weeks of training with partial squats [6]. Given the potential benefits of incorporating partial squats within resistance training programs, it is no surprise that these exercises are frequently prescribed [7,8,9,10].

A training variable that may be overlooked is the intent in which the prescribed exercises are performed. While multi-joint exercises, such as the back squat and bench press, may be implemented often within resistance training programs, whether they are performed in a ballistic (BAL; acceleration throughout the entire movement) or non-ballistic (NBAL; intentionally fast with a negative acceleration at the end of the movement) manner may produce different training adaptations. Previous research has examined the differences between BAL and NBAL upper and lower body exercises [11,12,13]. Newton et al. [11] indicated that performing bench press throws resulted in greater muscle activation, force production, velocity, and power output compared to traditional bench press repetitions performed at the same load. In contrast, Lake et al. [12] showed no difference in mean force or power between BAL and NBAL back squats; however, the authors did note that the method used to determine both variables affected the comparison. Further results from this study did show statistically significant differences in mean velocity and acceleration duration that favored back squats performed in a BAL manner. Similarly, Pestaña-Melero et al. [13] showed that BAL bench press repetitions produced greater velocities, while NBAL repetitions displayed greater force outputs. Despite the mixed evidence above, no research has compared the force-time characteristics of BAL and NBAL partial range of motion movements. To understand how to implement these exercises in training, further research is necessary.

The load prescribed during training may impact an exercise’s force production characteristics. For example, while most literature agrees that heavier loads may produce greater PF and impulse magnitudes [14,15,16,17,18,19], rapid force production characteristics may be emphasized using either lighter [15,20] and heavier [16,17] loads based on the exercise. Although an abundance of literature has examined the loading effects on exercises performed in a NBAL manner [14,21,22,23,24,25,26,27,28,29,30], an even greater amount of literature investigated how the external load affects the force-time characteristics of BAL exercises, such as jump squats [14,21,31,32,33,34,35], weightlifting movements and their derivatives [14,15,16,17,18,21,22,28,36,37,38,39,40,41,42,43,44,45,46,47], and bench press throws [19,27,35,48]. However, despite the abundance of BAL and NBAL loading literature, there is currently no research that examines how load affects the force-time characteristics of a partial range of motion squatting variation performed from a static starting position, such as the concentric-only half squat (COHS). Moreover, no literature has studied the differences in how load affects the force production characteristics of COHS performed with BAL and NBAL intent. To effectively prescribe partial squat variations, with BAL or NBAL intent, information about how load affects performance is essential. Therefore, the purpose of this study was to compare the PF and impulse characteristics of COHS performed in a BAL or NBAL manner. A secondary purpose was to examine how the external load affects the PF and impulse characteristics of BAL and NBAL COHS. It was hypothesized that BAL COHS would produce greater PF and impulse characteristics across all loads examined and that the external load would substantially impact the PF and impulse produced during COHS. It was also hypothesized that the greatest PF and impulse magnitudes would be produced at the heaviest loads.

## 2. Materials and Methods

### 2.1. Participants

Eighteen resistance-trained males (age = 24.4 ± 4.1 y, height = 179.2 ± 9.3 cm, body mass = 85.1 ± 9.2 kg, 1RM back squat = 161.1 ± 32.8 kg, relative 1RM back squat = 1.9 ± 0.3 kg/kg, 1RM COHS = 191.8 ± 29.7 kg, relative 1RM COHS = 2.3 ± 0.3 kg/kg) who were free of orthopedic injuries and regularly trained with the back-squat exercise (≥1 time per week for at least 6 months) volunteered to participate in this study. Each participant reported that they were performing at least three resistance sessions per week. Prior to testing, all participants read and provided written informed consent in accordance with the University’s Institutional Review Board.

### 2.2. 1RM Back-Squat Testing Session

A 1RM back-squat session was used to determine each participant’s 1RM back squat and determine the starting position and load estimation for the 1RM COHS testing session. Prior to testing, each participant completed a standardized warm-up protocol that started with two minutes of stationary cycling at 50 W at approximately 70 rpm. The cycling warm-up was followed by a dynamic warm-up that included a variety of dynamic stretches (e.g., forward walking lunge, straight leg march, etc.) covering 10 m and five repetitions each of slow bodyweight squats and fast bodyweight squats. Two minutes of rest were provided following the dynamic warm-up before the participant started the 1RM back-squat test protocol. Using a previously outlined protocol [49], each participant performed warm-up repetitions at 30, 50, 70, and 90% of their self-determined 1RM (5 at 30%, 5 at 50%, 3 at 70%, and 1 at 90%). Two-minute rest intervals were provided following the warm-up sets at 30 and 50% 1RM, while four minutes were provided after the warm-up sets at 70 and 90% 1RM. After the warm-up sets were completed, each participant completed maximal back-squat attempts at progressively increasing loads until a failed attempt occurred. Each 1RM attempt load was determined by the primary investigator and research assistants based on the previous 1RM attempt. A minimum 2.5 kg increase was required, and each participant achieved their 1RM back squat in four or fewer attempts. All back-squat repetitions were performed to a depth where the top of participant’s thigh was parallel to the floor. Squat depth was visually monitored during the 1RM testing by the principal investigator and research assistants. Four minutes of recovery was provided between each 1RM attempt.

Following the 1RM back-squat protocol, participants were provided with a self-selected rest period before assessing their 1RM COHS safety bar height. Each participant squatted with a 20 kg barbell to a knee angle of 90° and the safety bars of the squat rack were adjusted accordingly. After the safety bars were adjusted, the participant positioned themselves under the barbell and their knee angle was verified by using a handheld manual goniometer. The height of the safety bars was recorded, and the participant scheduled their 1RM COHS testing session.

### 2.3. 1RM Concentric-Only Half-Squat Testing Session

Participants returned for their 1RM COHS testing session one week following their 1RM back-squat session. The 1RM COHS testing session was used to determine the loads that would be used during the COHS testing sessions, and to familiarize the participants with the ballistic and non-ballistic testing conditions. Before maximal COHS attempts, participants performed the same warm-up protocol as described above. Similar to the 1RM back-squat testing session, the participant performed warm-up COHS repetitions at 30, 50, 70, and 90% of the participant’s estimated 1RM COHS (5 at 30%, 5 at 50%, 3 at 70%, and 1 at 90%) as described by previous research [49]. Previous pilot testing indicated that the 1RM COHS of participants was approximately 1.2× their 1RM back squat and the warm-up loads were calculated based on this information. After the final warm-up set, participants completed maximal COHS attempts at progressively increasing loads until a failed attempt occurred. Every COHS repetition was performed with the barbell resting on the safety pins of the squat rack with the participant starting with a 90° knee angle. The participants then extended their hips and knees using a concentric-only muscle action to finish each repetition [49]. Each participant achieved their 1RM COHS in four or fewer attempts with four minutes of recovery between each 1RM attempt.

### 2.4. Ballistic and Non-Ballistic Testing Sessions

**Figure 1:**
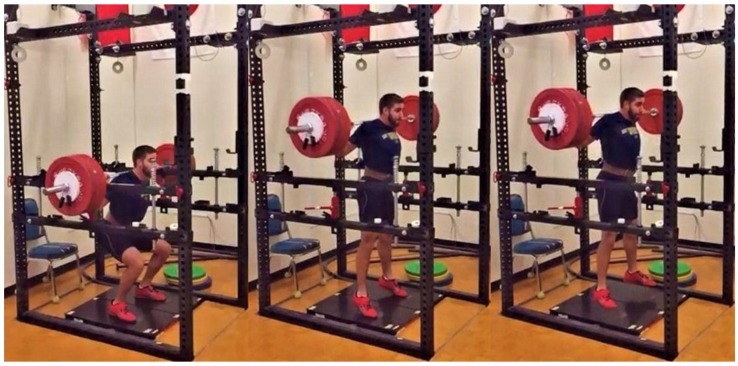
Ballistic concentric-only half-squat sequence. Note: The participant’s feet have lost contact with the force plates as a result of a jump.

**Figure 2:**
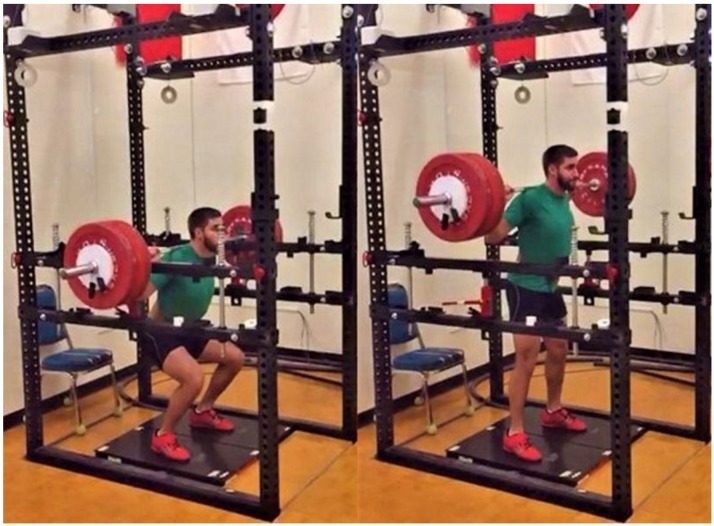
Non-ballistic concentric-only half-squat sequence. Note: The participant’s feet remained in contact with the force plates during the entire repetition.

One week following the COHS session, participants arrived for their BAL or NBAL COHS testing session. The order of these sessions was randomized and separated by 72–96 h to prevent an order effect. Similar to the 1RM sessions, participants began the COHS testing sessions by completing the same cycling and dynamic warm-up procedures outlined above. Following the standardized warm-up, each participant completed static squat jump repetitions at 50% and 75% of their perceived maximum effort before completing two maximal effort squat jumps with one minute of rest between jumps. Following the squat jump repetitions, the safety bars of the squat rack were adjusted to the participant’s designated height and the participant’s starting position was again confirmed using a handheld manual goniometer. Participants then received final instructions before performing their maximal effort BAL or NBAL COHS repetitions. Participants then performed COHS sets consisting of five repetitions at 30%, three repetitions at 50%, three repetitions at 70%, and two repetitions at 90% 1RM of their previously established 1RM COHS. While multiple repetitions were performed at each load, participants were instructed to “reset” between each repetition to ensure proper starting position and that the force-time record of each repetition was clearly separated from the others. The first two repetitions of each COHS set were used for statistical analyses. Two minutes of rest were provided following the sets at 30% and 50% 1RM and four minutes were provided following the set at 70% 1RM. Progressive sets with the given rest periods were completed to mimic how COHS may be used within resistance training sessions including warm-up repetitions. All COHS were performed in a BAL or NBAL manner as previously described [49,50,51]. Briefly, the participants were cued to “Stand up!” during the NBAL condition, while they were cued to “Finish!” (Onto the balls of their feet or jump if possible) during the BAL condition. Strong verbal encouragement was given during each repetition to ensure maximal effort. Figure 1 and Figure 2 display the start and finish of the BAL and NBAL conditions.

### 2.5. Data and Statistical Analyses

All COHS repetitions were performed on a dual force plate setup (2 separate 45.5 × 91 cm force plates; RoughDeck HP, Rice Lake, WI, USA) sampling at 1000 Hz. The COHS data were collected and analyzed using a customized LabVIEW program (2012 Version, National Instruments Co., Austin, TX, USA). A digital low-pass Butterworth filter with a cutoff frequency of 10 Hz was used to filter the voltage data obtained from the force plates to remove any noise from the signal. Relative PF was determined as the greatest instantaneous force value from the raw force-time data divided by the participant’s body mass. Impulse data from 0–50 ms (Imp50), 0–90 ms (Imp90), 0–200 ms (Imp200), and 0–250 ms (Imp250) were calculated as the product of force and time from the onset of the COHS repetition to the designated time intervals above. These time intervals were chosen based on their relationships with striking [52], sprinting [53], and jumping [54]. Impulse data was then divided by the participant’s body mass to determine relative impulse. The onset of each repetition was determined using visual inspection of the force-time [55]. Each COHS repetition was used for the reliability analysis and the two-repetition average PF, Imp50, Imp90, Imp200, and Imp250 data were used for further statistical analysis.

Normality of the data was assessed using a Shapiro-Wilk test. Two-way mixed intraclass correlation coefficients (ICC) and typical error (TE) expressed as a coefficient of variation percentage were used to assess test-retest reliability of each variable. A series of 2 × 4 (Intent × Load) repeated measures ANOVAs were used to examine the differences in relative PF, Imp50, Imp90, Imp200, and Imp250 between BAL and NBAL COHS performed at relative loads of 30, 50, 70, and 90% 1RM COHS. If the assumption of sphericity was violated, Greenhouse-Geisser adjusted values were reported. Statistical power (c) was calculated for all main effect comparisons. Cohen’s d effect sizes were calculated to provide a measure of practical significance and were interpreted based on the scale discussed by Hopkins [56]. Finally, 95% confidence intervals (CI) were calculated for all pairwise comparisons. All statistical analyses were performed using SPSS 25 (IBM, Armonk, NY, USA). A Bonferroni correction was used to correct for Type I error, where the original criterion *p*-value of 0.05 was divided by five based on the number of repeated measures ANOVA tests. Therefore, statistical significance was determined as *p* ≤ 0.01.

## 3. Results

All data were normally distributed. The reliability and descriptive statistics for PF, Imp50, Imp90, Imp200, and Imp250 at each load examined for BAL and NBAL COHS are displayed in Table 1 and Table 2, respectively.

### 3.1. Intent Effects

Statistically significant main effect differences existed PF (*p* < 0.001, c = 1.00). Post hoc analysis indicated that load-averaged BAL COHS produced greater PF magnitudes compared to NBAL COHS (*p* < 0.001, d = 1.30, CI = 6.05–8.80). In addition, statistically significant main effect differences existed for Imp90 (*p* = 0.006, c = 0.84), Imp200 (*p* = 0.001, c = 0.96), and Imp250 (*p* < 0.001, c = 0.98), but not for Imp50 (*p* = 0.018, c = 0.69) Post hoc analysis indicated that load-averaged BAL COHS produced statistically greater Imp90 (d = 0.25, CI = 0.02–0.11), Imp200 (d = 0.36, CI = 0.11–0.38), and Imp250 (d = 0.41, CI = 0.19–0.55) compared to NBAL COHS.

### 3.2. Load Effects

Statistically significant main effect differences were found between intent-averaged loads for COHS PF, Imp50, Imp90, Imp200, and Imp250 (all *p* < 0.001, c = 1.00). Moderate-large effect sizes existed across loads for PF (d = 0.77–2.38), while small-moderate effect sizes existed across loads for Imp50 (d = 0.20–0.92), Imp90 (d = 0.21–0.96), Imp200 (d = 0.21–1.00), and Imp250 (d = 0.20–1.08).

### 3.3. Intent x Load Interaction Effects

**Figure 3:**
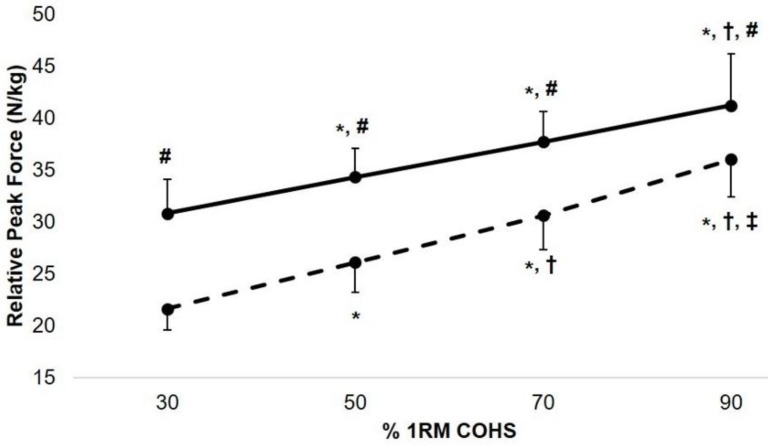
Intent and load interactions for relative peak force during ballistic (solid line) and non-ballistic (dashed line) concentric-only half-squats (COHS) performed with 30, 50, 70, and 90% one repetition maximum (1RM) COHS. * = statistically greater than 30% 1RM (*p* < 0.05); † = statistically greater than 50% 1RM (*p* < 0.01); ‡ = statistically greater than 70% 1RM (*p* < 0.001); # = statistically greater than non-ballistic condition (*p* < 0.001).

**Figure 4:**
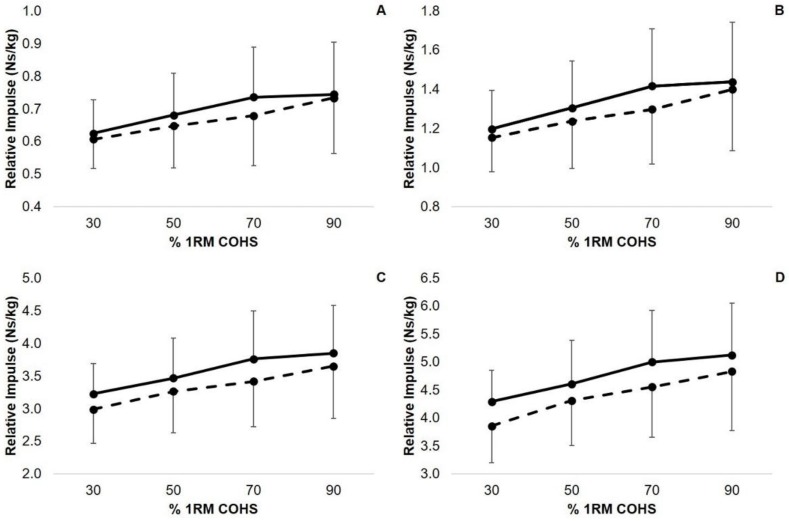
Intent and load interactions for relative impulse produced at 50 ms (**A**), 90 ms (**B**), 200 ms (**C**), and 250 ms (**D**) during ballistic (solid line) and non-ballistic (dashed line) COHS performed with 30, 50, 70, and 90% one repetition maximum (1RM) COHS.

A statistically significant intent x load interaction effect existed for COHS PF (*p* < 0.001, c = 1.00). Post hoc analysis indicated that BAL COHS produced statistically greater PF than NBAL COHS at 30% (*p* < 0.001, d = 3.37), 50% (*p* < 0.001, d = 2.88), 70% (*p* < 0.001, d = 2.29), and 90% 1RM (*p* < 0.001, d = 1.19). In contrast, there were no statistically significant intent x load interaction effects for Imp50 (*p* = 0.263, c = 0.34), Imp90 (*p* = 0.341, c = 0.29), Imp200 (*p* = 0.509, c = 0.15), or Imp250 (*p* = 0.493, c = 0.14). Figure 3 and Figure 4 display the intent and load interactions for PF and Imp50, Imp90, Imp200, and Imp250 during both the BAL and NBAL conditions, respectively.

## 4. Discussion

This study compared the differences between COHS performed in a BAL or NBAL manner and the effect of load on PF and impulse characteristics. The findings of the current study support our hypotheses. BAL COHS produced statistically greater PF compared to NBAL COHS. Load-averaged BAL COHS produced statistically greater Imp90, Imp200, and Imp250 compared to NBAL COHS, while no difference existed for Imp50. Finally, statistically significant differences existed between loads for intent-averaged COHS for all the examined variables, with the heaviest loads resulting in the greater magnitudes of PF and impulse.

Very large effect sizes existed when comparing the PF between BAL and NBAL COHS [56]. Similar to previous findings [11], but in contrast to others [12,13], the BAL COHS in the current study produced greater PF magnitudes compared to the NBAL COHS at each of the relative loads examined. These findings may be based on the physiological nature of BAL exercise. Previous research has indicated that BAL exercise may lower the recruitment threshold of motor units [57,58], and allow the motor neuron pool to be fully activated within milliseconds [59], ultimately resulting in greater force production. Thus, it should come as no surprise that the heaviest loads used during the COHS (90% 1RM or 108.6 ± 9.6% of the participants’ 1RM back squat) combined with BAL intent produced the greatest PF magnitude. An interesting finding of the current study is that the largest effect size magnitudes for PF between BAL and NBAL COHS existed at the lightest load (30% 1RM), while the smallest, albeit still large effect, existed at the heaviest load (90% 1RM). This finding may be explained by the ability of an individual to develop high rates of force development with lighter loads during BAL exercises [15]. In addition, it is possible that the lighter loading conditions required participants during the NBAL condition to stop applying force earlier to maintain contact with force plates. This notion may be supported by the non-statistically significant Imp50 findings and statistically significant Imp90, Imp200, and Imp250 findings. Although rate of force development was not compared in the current study, the impulse characteristics of the current study support this notion. From practical standpoint, these findings show that light and heavy training loads should still be moved with maximal intent to receive the greatest training stimulus. Thus, it may be beneficial to cue athletes to move every load with maximal intent during the concentric portion of the movement (e.g., warm-up sets, working sets, and warm-down/drop sets).

The impulse generated by an individual may ultimately determine the performance of specific tasks, such as vertical jump and weightlifting movements [60]. Except for Imp50, the findings of the current study indicated that when averaged across loads, BAL COHS produced greater impulse magnitudes compared to NBAL COHS. Although only small effects were predominantly present, it should be noted that the practical differences increased as the time interval increased. Given that the participants in the current study had to perform each COHS repetition from a static starting position and without the benefit of a stretch-shortening cycle, the length of time for each repetition was considerably longer than the time intervals examined. While the time intervals examined in the current study are related to striking [52], sprinting [53], and jumping [54] tasks, the early force production characteristics of BAL and NBAL COHS cannot be overlooked. Specifically, the differences in early force characteristics during the BAL condition may ultimately culminate in larger peak force production, and greater forces throughout the movement, which is supported by our results.

Similar to the PF results, the heaviest load examined produced the largest impulse magnitudes at each of the examined time intervals. However, in contrast to the PF results, only small-moderate effect size magnitudes existed between loads. These findings may be due to the large variation in relative impulse that was present among the participants at each time interval. A recent review discussed the influence that muscular strength can have on general and specific sport performance tasks; however, the authors also discussed how greater muscular strength may positively influence rapid force production characteristics [61]. The relative 1RM back squat and COHS of the participants in the current study ranged from 1.4–2.4 and 1.8–2.7 times their body weight, respectively. Although the effects of strength on early impulse qualities was not examined in the current study, the wide range of relative strength characteristics may have resulted in a larger variation between the participants. Future research may consider investigating the force-time characteristics of BAL and NBAL COHS between stronger and weaker individuals.

A potential limitation to the current study may have been the non-randomization of load order. Although the likelihood is small based on the single set performed at each load with five or fewer repetitions, it is possible that some of the weaker participants fatigued during the progressive sets, which may have negatively impacted their performance at heavier loads. However, as noted above, the progressive sets used within this study provide a good representation of how COHS may be used within an actual training setting (e.g., warm-up sets followed by working sets). Future research may consider examining the performance differences between stronger and weaker individuals during BAL and NBAL COHS as well as other full and partial range of motion exercises.

## 5. Conclusions

COHS performed with BAL intent produced greater relative PF and impulse magnitudes compared to COHS performed with NBAL intent. Greater force and impulse magnitudes (except for Imp50) may be achieved at early time intervals and maintained throughout a COHS by performing the exercise with BAL intent. The external load used during BAL and NBAL COHS may have a moderate to large practical effect on PF and a small-moderate effect on impulse magnitudes produced at various time intervals.

BAL COHS produced larger PF and impulse magnitudes at several relative loads. Thus, performing COHS with BAL intent may provide a favorable training stimulus compared to COHS performed with NBAL intent, especially when performed at lighter loads. Thus, athletes should be cued to move the external load as fast as possible during the concentric phase of the movement to receive the greatest training stimulus during all exercise sets (e.g., warm-up, working, and warm-down/drop sets). Given the reduced range of motion, potential use of heavier loads (>1RM back squat), and BAL intent, BAL COHS may be best implemented during resistance training phases in which the goal is to develop maximal force production, impulse, and rate of force development characteristics.

## Figures and Tables

**Table 1:** Reliability statistics of ballistic and non-ballistic concentric-only half-squat repetitions.

Load (% 1RM COHS)	PF		Imp50		Imp90		Imp200		Imp250	
	ICC	TE%	ICC	TE%	ICC	TE%	ICC	TE%	ICC	TE%
Ballistic										
30%	0.95	5.7	0.94	7.2	0.94	7.3	0.90	9.4	0.90	8.9
50%	0.96	4.2	0.97	7.0	0.97	7.4	0.95	8.4	0.95	8.0
70%	0.98	2.7	0.95	7.4	0.95	7.8	0.95	8.9	0.95	8.3
90%	0.90	6.7	0.92	12.2	0.92	13.1	0.88	16.0	0.87	16.4
Non-ballistic										
30%	0.95	3.9	0.93	7.4	0.93	7.5	0.95	7.7	0.95	7.5
50%	0.99	2.4	0.98	6.8	0.97	7.5	0.95	10.3	0.95	10.8
70%	0.98	2.8	0.94	9.6	0.94	9.4	0.95	8.7	0.95	8.7
90%	0.98	2.5	0.98	6.0	0.97	6.4	0.95	7.8	0.95	8.4

Notes: 1RM COHS = one repetition maximum concentric-only half squat; PF = relative peak force; Imp50 = relative impulse from 0–50 ms; Imp90 = relative impulse from 0–90 ms; Imp200 = relative impulse from 0–200 ms; Imp250 = relative impulse from 0–250 ms; ICC = intraclass correlation coefficient; TE% = typical error expressed as a coefficient of variation percentage.

**Table 2:** Descriptive statistics of ballistic and non-ballistic concentric-only half-squat repetitions.

Load (% 1RM COHS)	PF †‡ (N/kg)	Imp50 ‡ (Ns/kg)	Imp90 †‡ (Ns/kg)	Imp200 †‡ (Ns/kg)	Imp250 †‡ (Ns/kg)
Ballistic					
30%	30.8 ± 3.3	0.62 ± 0.1	1.20 ± 0.2	3.22 ± 0.5	4.29 ± 0.6
50%	34.3 ± 2.8	0.68 ± 0.1	1.30 ± 0.2	3.47 ± 0.6	4.61 ± 0.8
70%	37.7 ± 2.9	0.73 ± 0.2	1.42 ± 0.3	3.76 ± 0.7	5.00 ± 0.9
90%	41.2 ± 5.0	0.74 ± 0.2	1.44 ± 0.3	3.85 ± 0.7	5.12 ± 0.9
Non-ballistic					
30%	21.6 ± 2.0	0.61 ± 0.1	1.15 ± 0.2	2.99 ± 0.5	3.86 ± 0.7
50%	26.1 ± 2.9	0.65 ± 0.1	1.24 ± 0.2	3.26 ± 0.6	4.31 ± 0.8
70%	30.6 ± 3.3	0.68 ± 0.2	1.30 ± 0.3	3.42 ± 0.7	4.55 ± 0.9
90%	36.0 ± 3.6	0.73 ± 0.2	1.40 ± 0.3	3.65 ± 0.8	4.83 ± 1.1

Notes: 1RM COHS = one repetition maximum concentric-only half squat; PF = relative peak force; Imp50 = relative impulse from 0–50 ms; Imp90 = relative impulse from 0–90 ms; Imp200 = relative impulse from 0–200 ms; Imp250 = relative impulse from 0–250 ms; † = statistically significant intent main effect (*p* < 0.01); ‡ = statistically significant load main effect (*p* < 0.001).
